# Study progress, recreational activities, and loneliness in young adult carers: a national student survey

**DOI:** 10.1186/s40359-022-00760-5

**Published:** 2022-02-26

**Authors:** Bente Storm Mowatt Haugland, Mari Hysing, Børge Sivertsen

**Affiliations:** 1 Department of Clinical Psychology, Faculty of Psychology, University of Bergen, Årstadveien 17, 5009 Bergen, Norway; 2 Department of Psychosocial Science, Faculty of Psychology, University of Bergen, Bergen, Norway; 3 Department of Health Promotion, Norwegian Institute of Public Health, Bergen, Norway; 4 Department of Mental Health, Norwegian University of Science and Technology, Trondheim, Norway; 5 Department of Research & Innovation, Helse Fonna HF, Haugesund, Norway

**Keywords:** Young adult carers, National student survey, Study progress, Loneliness, Recreational activities

## Abstract

**Background:**

Young adults (18–25 years) with informal care responsibilities have received limited attention in the research literature, and little is known on how caring responsibilities are related to functioning across different life domains. In the present study we examine associations between care responsibilities and study progress, recreational life, and loneliness in young adults in higher education.

**Methods:**

A national survey was conducted among Norwegian students in higher education (the SHoT2018-study). The response rate was 30.8%. The current sample is a subsample of the respondents, including young adults 18 to 25 years old, comprising 40.205 participants (70.2% women, mean age 22.0 years, SD = 1.7). Participants reported whether they had regular care responsibility for someone with physical or mental illness, disabilities, or substance misuse. They also answered questions on study progress, number of hours studying, physical exercise, involvement in organized volunteer student activities, number of close friends, and feelings of loneliness. Data were analyzed by Chi-square tests and logistic regression analyses, adjusting for age, sex, and chronic illness.

**Results:**

Compared to students without care responsibility, young adult carers (*n* = 2228, 5.5% of study sample) were more likely to report delayed study progress (OR 1.20, *p* < .001), higher average number of failed exams (e.g., having failed three times or more, OR 1.31, *p* = .002), more feelings of loneliness (OR 1.26, *p* < .001), and slightly fewer friends. Those with limited care responsibility (≤ 1 h daily) were more likely to participate in organized volunteer student activities, whereas students with 2 h or more of caring per day were less likely to participate in leisure student activities. Both study progress and feelings of loneliness were related to care responsibility in a response-dose pattern, with worse outcomes for those with 2 h or more of daily caring responsibility. All comparisons were adjusted for age, sex, and chronic illness.

**Conclusions:**

Study progress, recreational activities, and loneliness among young adults are associated with informal caring responsibilities. Professionals in the educational system as well as health personnel should be sensitized to the needs of young adult carers and necessary support made available.

## Background

During the last 25 years research studies have examined different aspects of how youth below 18 years of age are affected by informal care responsibility [1]. However, we have considerably less research evidence about young adults above 18 years who provide informal care [2–5]. Young adult carers (YACs) have been defined as individuals between 18 and 25 years of age who provide informal care to one or more family member, relative, or other, due to physical or mental illnesses, substance abuse, or disabilities in the care-recipient [6]. The caring undertaken by YACs may comprise a range of different responsibilities, including household tasks (e.g., cooking, cleaning), emotional care (e.g., supporting, supervising), practical support (e.g., paying bills, administering medication), or personal care (e.g., bathing, dressing) [6]. We need to expand our knowledge about YACs, as this is a group expected to grow in number due to an aging population in western societies, and increased reliance on outpatient treatment and home-based care [7].

Caring for family members or others has been described as rewarding and meaningful for young caregivers [8]. The psychological benefits may include developing a close relationship with the cared-for individual, enhanced feelings of purpose, meaning, and pride, acquisition of competence and social skills, and a sense of maturity and personal growth [3, 6, 9, 10]. However, for YACs also increased levels of mental and somatic health problems have been observed [4, 6, 9, 11–14]. We have previously compared Norwegian students (N = 40 205) with and without care responsibility for family members or others with physical or mental illness, disabilities, or substance misuse. The results showed that students with care responsibilities (5.5% of the total sample) reported higher levels of mental health problems (i.e., anxiety and depression), more insomnia and somatic symptoms (e.g., headaches, backpain), as well as lower life satisfaction compared to students without care responsibilities. Furthermore, the number of hours spent on caring was associated with negative health outcomes in a dose response pattern [15]. In the present study we expand on these findings by examining the consequences of informal caring for the study progress, recreational activities, and feelings of loneliness in the same population-based national sample of young adult students.

Whereas previous studies have indicated that caregiving may restrict school engagement, educational aspirations, and choices, as well as the social life of young carers below 18 years [10, 16, 17], very few have examined the educational, recreational, and social consequences of caring for YACs [4]. This is unfortunate, as it has been suggested that caring may have different impact on education, employment, social life, and well-being of young carers below and above 18 years [18].

Young adulthood is a life phase when significant choices are made that may decide the education and future career of the young person. For many, this is a period when educational attainments are of particular importance. Also, voluntary relationships (i.e., friends and romantic partners) that may form the future identity of the young adult are explored [19]. Thus, the years between 18–25 have been described as a period of transition and identity formation, being “no longer adolescent but only partly adult” [19]. Young adults are expected to leave home, establish committed and intimate relationships, and engage in social, academic, and recreational activities independent of their family of origin [19]. With these developmental tasks in mind, it is important to understand what consequences informal care responsibilities may have on the education and recreational life of young adults.

A previous study, applying qualitative interviews (N = 36), found that the struggle to find a balance between fulfilling care responsibilities, having time for personal and social activities, and pursuing educational aims was common for young cares as well as for YACs [18]. However, whereas some YACs found it challenging to combine care responsibilities and studies, others appreciated the more flexible time-schedule offered in higher education, giving them the opportunity to look after the care-recipient during the day when necessary [18]. Furthermore, only a minority of the YACs were satisfied with their social lives, reporting that the caring role took priority over recreational activities and seeing friends.

Educational, social, and recreational consequences for YACs were examined in a pioneer study by Becker and Becker (2008). They interviewed 25 YACs (16–25 years) and found that many experienced restricted opportunities for social life and leisure activities due to lack of time and money. Whereas some had positive experiences from attending college, others left higher education prematurely, partly because of competing demands between caring tasks and studies [6]. These findings were supported in a later survey (N = 295, 14–25 years) where 55% of the carers reported that the care responsibility made it difficult to attend college or university, and 17% were concerned that the caring would cause them to drop out [11]. Furthermore, a substantial number of days were lost to absence due to care responsibilities. However, a large percentage of the carers (79%) enjoyed attending higher education, and 70 percent thought they were doing well.

According to Vasileiou, Barnett [20] a restricted social life, little time for friendship, and high levels of loneliness are commonly found among informal caregivers of different age groups (range 24–91 years). While little empirical data are found on the social life and loneliness among YACs in particular, social support has been related to positive outcomes in young Australian carers (N = 100, 10—25 years) [21].

A literature review on YACs suggests that even though the caregiving role may provide a short-time sense of security and involvement, it may also represent a barrier to education and reduced engagement in studies and in social participation [2]. Whereas some studies report associations between informal caring and mental health outcomes [12, 15], we need to broaden the perspective to also examine other domains in the life of a young person that may be affected when time, energy, and attention is invested in caring. This may specially be the case for young adults who are in a life-course stage where higher education, career choice, establishing a social life, and leaving the family home is in focus [18]. Issues related to education, social relations, and leisure activities are key areas of life, important to include when comparing young adults with and without caring responsibilities. In addition to other issues (e.g., establishing economic independence, well-being, and self-efficacy) these are life-domains regarded as essential for the understanding of how caring responsibilities may affect the lives of YACs. Furthermore, examining these domains may increase our awareness of the needs for support among YACs.

Many previous studies on YACs apply qualitative methods (e.g., 6, 18, 22, 23). These studies offer detailed descriptions and insights regarding the experiences and needs of YACs. However, the generalizability of the findings is limited due to small sample sizes and frequent recruitment of responders from interest organizations (e.g., 6, 10, 18, 24, 25). Thus, a recent review of research studies concludes that well-designed research is highly needed, including larger, representative samples of young carers 18 to 25 years, as well as control groups [4].

Summing up, being an informal caregiver may be demanding and placing a young person under considerable stress [9, 26, 27]. A recent study found that half of students (16–25 years) who grew up with a chronically ill family member reported that the illness influenced their daily life, albeit to what extent and in what domains was not specified [28]. In the present study we aim to examine associations between care responsibility and specific and highly relevant domains of the everyday life of young adults: study progress, recreational life, and loneliness. The first research question concerns study progress and time spent on studies, comparing young adults with and without care responsibilities. Secondly, we examine whether recreational activities (e.g., physical exercise, participation in sports, cultural activities, and voluntary student activities) differ for YACs compared to young adult without care responsibilities. Thirdly, we look at feelings of loneliness among young adults with and without care responsibilities, and finally, we examine associations between the number of hours spent on caring and study progression, recreational activities, and loneliness.

## Method

### Procedure

The SHoT2018 study (Students’ Health and Wellbeing Study) is a national student survey for higher education in Norway, initiated by the three largest student welfare organizations [Sammen (Bergen and surrounding area), Sit (Trondheim and surrounding area), and SiO (Oslo and Akershus)]. In the SHoT2018 study, data were collected electronically through a web-based platform. Details of the study have been published elsewhere (Sivertsen et al., 2019a), but in short, the SHoT2018 was conducted between February 6 and April 5, 2018, and invited all fulltime Norwegian students pursuing higher education (both in Norway and abroad) to participate. In all, 162,512 students fulfilled these inclusion criteria, of whom 50,054 students completed the online questionnaires, yielding a response rate of 30.8%. As the current study was an investigation of YACs, we excluded participants aged 26 years and older, yielding a final sample size of 40,205 participants, aged 18–25 years. The average time spent answering the questionnaire was 21 min. Although a few universities and colleges allocated time in school classes allowing the student to complete the survey during a lecture, no teachers were instructed to provide support or assistance.

### Instruments

#### Sociodemographic factors and self-reported illness

All participants indicated their age and sex, and participants were also asked about their relationship status and if they had children of their own. Participants were categorized as immigrants if either the student or one or both of his/her parents were born outside Norway.

Self-reported illnesses and disorders were assessed by a pre-defined list adapted to fit this age-cohort. The list was based on a similar operationalization used in previous large population-based studies (the HUNT study [29]) and included several subcategories for most conditions/disorders (not listed here). For somatic illnesses, the list comprised the following specific illnesses/group of illnesses: cerebral paresis, epilepsy, fibromyalgia, heart disease, cancer, myalgic encephalomyelitis/chronic fatigue syndrome (ME/CFS), rheumatoid arthritis, and multiple sclerosis (MS). Participant also indicated if they had a mental disorder. The list contained no definition of the included disorders/conditions. All participants were also asked how old they were when they got the illness/disorder. For purposes of the current study, “chronic illness” was defined as having had one of the above somatic illnesses or a mental disorder for at least 5 years.

#### Exposure variable

All students were asked if they had regular care responsibilities for someone with physical or mental illness, disabilities, or substance misuse (not his/her own child/children). If answering yes to this question, they were asked how many hours they spent on a typical weekday and weekend day to help this person(s). The exact phrasing of the questions is detailed in Table 1. These were survey questions that have previously been tested for clarity among young carers (5–17 years) and their parents   [8].

**Table 1:** Questions used to assess care responsibilities

Some people provide help or support to people who are physically or mentally ill, disabled or misusing drugs or alcohol. This could be a parent, brother, sister, another relative or someone else. Is there anyone like this who you have to look after on an ongoing basis?	
□ Yes, someone I live with	
□ Yes, someone I do not live with	
□ No	
*If Yes:*	
About how many hours do you spend on a typical weekday to help this person(s)?	
About how many hours do you spend per day on weekends / vacations to help this person(s)?	

Reprinted from Haugland, Hysing and Sivertsen (2020), Frontiers in Psychology, https://doi.org/10.3389/fpsyg.2021.638879

#### Study progress

Self-reported study progress was assessed with the following two questions:” Are you following your normed study progression (30 credits per semester) on the study program you are taking now?” with the response options” yes” and “no”; and ”Have you failed an exam after you started studying at your college/university?” with the response options”yes” and “no”. A normed study progression for a full-time student in Norway means obtaining 30 credits per semester. Those who, for various reasons, obtain less than 30 credits per semester have a delayed study progress and risk having to spend an extra semester (or a whole school year) to complete their degree. Passed exams give study credits and a failed exam can result in delayed study progress. Some courses offer the option to retake the exam shortly after failing an examination, which prevents delayed study progress for those who pass the re-take exam.

The number of exams per semester can vary greatly and can result in that the number of exams per semester may vary greatly across study programs. Information about the number of exams the students had per semester was not available in this study. The above descripton of the study progress measure is derived from a previous study where the identical measure was applied [30].

In addition to the assessment of study progress, all students were asked how many hours they spend studying last week (including classes and self-study).

#### Recreational activities

Recreational activities were measured by level of physical activity and involvement in organized volunteer student activities (e.g., cultural activities, interests societies).

Physical exercise was assessed using three sets of questions, assessing the average number of times exercising each week, and the average intensity and average hours each time [31]: (1) “How frequently do you exercise?” (Never, Less than once a week, Once a week, 2–3 times per week, Almost every day); (2) “If you do such exercise as frequently as once or more times a week: How hard do you push yourself? (I take it easy without breaking into a sweat or losing my breath, I push myself so hard that I lose my breath and break into a sweat, I push myself to near-exhaustion); and (3) “How long does each session last?” (Less than 15 min, 15–29 min, 30 min to 1 h, More than 1 h). Based on international recommendation that adults should get at least 30 min of moderate to vigorous physical activities (MVPA) per day or more per week (= 150 min per week), [32] we created a variables for which students answering both “Almost every day” of the frequency item, “I push myself so hard that I lose my breath and break into sweat” on the intensity item, and “30 min or more” on the duration item, were coded as meeting the recommendations of “MVPA: 150 min/week”.

In addition, all students were asked if they were involved in the following organized volunteer student activities: sports, cultural activities, student democracy, professional societies, or other interests societies.

#### Loneliness and number of close friends

Loneliness was assessed using an abbreviated version of the widely used UCLA Loneliness Scale, “The Three-Item Loneliness Scale (T-ILS)”. The T-ILS include the following three items, each rated along a 5-point Likert scale (“never”, “seldom”, “sometimes”, “often”, and “very often”) [33]. For each question below, please indicate how often you have felt that way during the last year: (1) How often do you feel that you lack companionship? (2) How often do you feel left out, and (3) How often do you feel isolated from others? The T-ILS has displayed satisfactory reliability and both concurrent and discriminant validity [33]. In the current study, the T-ILS was used both as a continuous total score, and as three dichotomous variables comparing “never”, “seldom”, “sometimes”, versus “often”, and “very often” for each item.

All students were also asked how many friends they have that they are close to or can talk to about different problems.

### Statistics

IBM SPSS version 27 (SPSS Inc., Chicago, IL, United States) for Windows was used for all analyses. Chi-square tests were used to examine possible demographical differences (sex, age, ethnicity, having children, and marital status) and chronic illness between students with care responsibilities and the control group (students with no care responsibilities). Chi-square tests were also used to investigate the association between care responsibilities and study progress, participation in student activities, and loneliness. Logistic regression analyses were conducted to provide effect-size estimates [odds-ratios (ORs)], adjusting for age, sex, and chronic illness. We tested for pairwise comparisons of proportions across outcomes between groups of hours of care responsibilities by employing the “Compare column proportions” function available for Chi-square tests in SPSS.

The normality of the data was examined using skewness and kurtosis, and all continuous measures were well within the recommended ranges (± 2) [34]. There was generally little missing data, and hence missing values were handled using listwise deletion. As the SHoT2018 study had several objectives and was not designed to be a study of students with care responsibilities specifically, no a priori power calculations were conducted to ensure that the sample size had sufficient statistical power to detect differences in outcomes.

## Ethics

The SHoT2018 study was approved by the Regional Committee for Medical and Health Research Ethics in Norway (no. 2017/1176). An electronic informed consent was obtained after the participants had received a detailed introduction to the study.

## Results

### Sample characteristics

The sample was drawn from a national survey in Norway from 2018 among students in higher education (the SHoT2018-study), comprising 40,205 participants, 70.2% women, mean age 22 years (SD = 1.7). As detailed in Table 2, having care responsibilities for others was more common among female students compared to male students (6.4 versus 3.5%, respectively). Furthermore, having care responsibilities for others was associated with not being single, having own children, being of non-Norwegian ethnicity, and reporting more chronic illness. Among those reporting care responsibility, mean hours of daily care was 1.8 h (SD 2.1) on weekdays and 3.7 h (SD 3.3) on weekend-days.

**Table 2:** Descriptive characteristics by care responsibilities of others

Sociodemographic factors	Care responsibilities				
	No (94.5%, n = 37,977)		Yes (5.5%, n = 2228)		*p*-value^$^
	%	(n)	%	(n)	
Age, mean (SD)	22.0	(1.73)	22.1	(1.77)	ns
Sex, % (n)					< .001
Females	93.6%	[26, 324]	6.4%	(1804)	
Male	96.5%	[11, 521]	3.5%	(416)	
Ethnicity, % (n)					< .001
Ethnic Norwegian	94.6%	[35, 127]	5.4%	(2011)	
Immigrant	92.9%	(2850)	7.1%	(217)	
Own children, % (n)					.001
Yes	90.8%	(405)	9.2%	(41)	
No	94.5%	[37, 470]	5.5%	(2176)	
Marital status, % (n)					< .001
Not single	96.9%	[17, 767]	6.1%	(1157)	
Single	95.0%	[20, 154]	5.0%	(1068)	
Chronic illness, % n)					< .001
No	95.2%	[34, 475]	4.8%	(1743)	
Yes	87.8%	(3502)	12.2%	(485)	

^$^ = *p*-values are based on the Chi-squared analyses and adjusted for multiple comparisons using the Benjamini–Hochberg correction

### Study progress

As detailed in Table 3, YACs study progress was poorer among students with care responsibility compared to students without care responsibilities. While 17.3% of students without care responsibilities were delayed in their study progression, the corresponding proportion among YACs was 21.4% (*p* < 0.001). YACs also had more failed exams than the control group, with 37.5% of the YAC group having failed one or more exams, compared to 31.2% in the control group. (*p* < 0.001). Furthermore, as detailed in Table 3, YACs changed they study program more often than students without care responsibilities. However, there were no significant group differences between students with and without care responsibility in terms of hours spent studying per week (see Table 3 for details).

**Table 3:** Study progress and loneliness by care responsibilities of others

	Care responsibilities						
	No (94.5%, n = 37,977)		Yes (5.5%, n = 2228)		*p*-value^$^	Adjusted OR^#^	
*Study progress*							
Delayed study progression, % (n)	17.3%	(6565)	21.4%	(476)	< .001	1.20	(1.02–1.41)
Number of failed exams, mean (SD)	0.66	(1.36)	0.79	(1.42)	< .001	n/a	
Never, % (n)	68.8%	[26, 132]	62.5%	(1393)	.001	1.00	-
Once, % (n)	15.9%	(6023)	18.6%	(414)	.001	1.27	(1.13–1.42)
Twice, % (n)	7.1%	(2701)	8.8%	(197)	.002	1.35	(1.16–1.58)
Three times or more	8.2%	(3121)	10.1%	(224)	.002	1.31	(1.13–1.52)
Times changed your study program, mean (SD)	0.33	(0.58)	0.39	(0.63)	< .001	n/a	
Never, % (n)	72.6%	[27, 040]	68.4%	(1485)	< .001	1.00	-
Once, % (n)	21.8%	(8124)	23.7%	(515)	.037	1.18	(0.99–1.42)
Two or more, % (n)	5.6%	(2081)	7.9%	(171)	< .001	1.27	(1.07–1.51)
Hours spent on studies per week, mean (SD)	24.0	(15.4)	23.9	(16.8)	Ns	n/a	
*Loneliness items*							
T-ILS 1: Lack companionship*, % (n)	22.8%	(8638)	28.4%	(628)	< .001	1.18	(1.07–1.30)
T-ILS 2: Left out*, % (n)	16.2%	(6110)	23.5%	(520)	< .001	1.32	(1.19–1.46)
T-ILS 3: Isolated*, % (n)	15.6%	(5855)	23.4%	(515)	< .001	1.37	(1.23–1.52)
T-ILS: All three items *, % (n)	5.3%	(1901)	8.0%	(309)	< .001	1.26	(1.11–1.44)
T-ILS: Total loneliness score, mean (SD)	7.4	(3.0)	8.2	(3.2)	< .001	n/a	
Number of close friends, mean (SD)	3.6	(0.9)	35	(0.9)	< .001	n/a	

^*^ = % reporting “often or very often”

^$^ = *p*-values are based on the Chi-squared analyses and adjusted for multiple comparisons using the Benjamini–Hochberg correction

^#^ = Adjusted for age, sex, and chronic illness

### Physical exercise and participation in  recreational activities

**Fig. 1:**
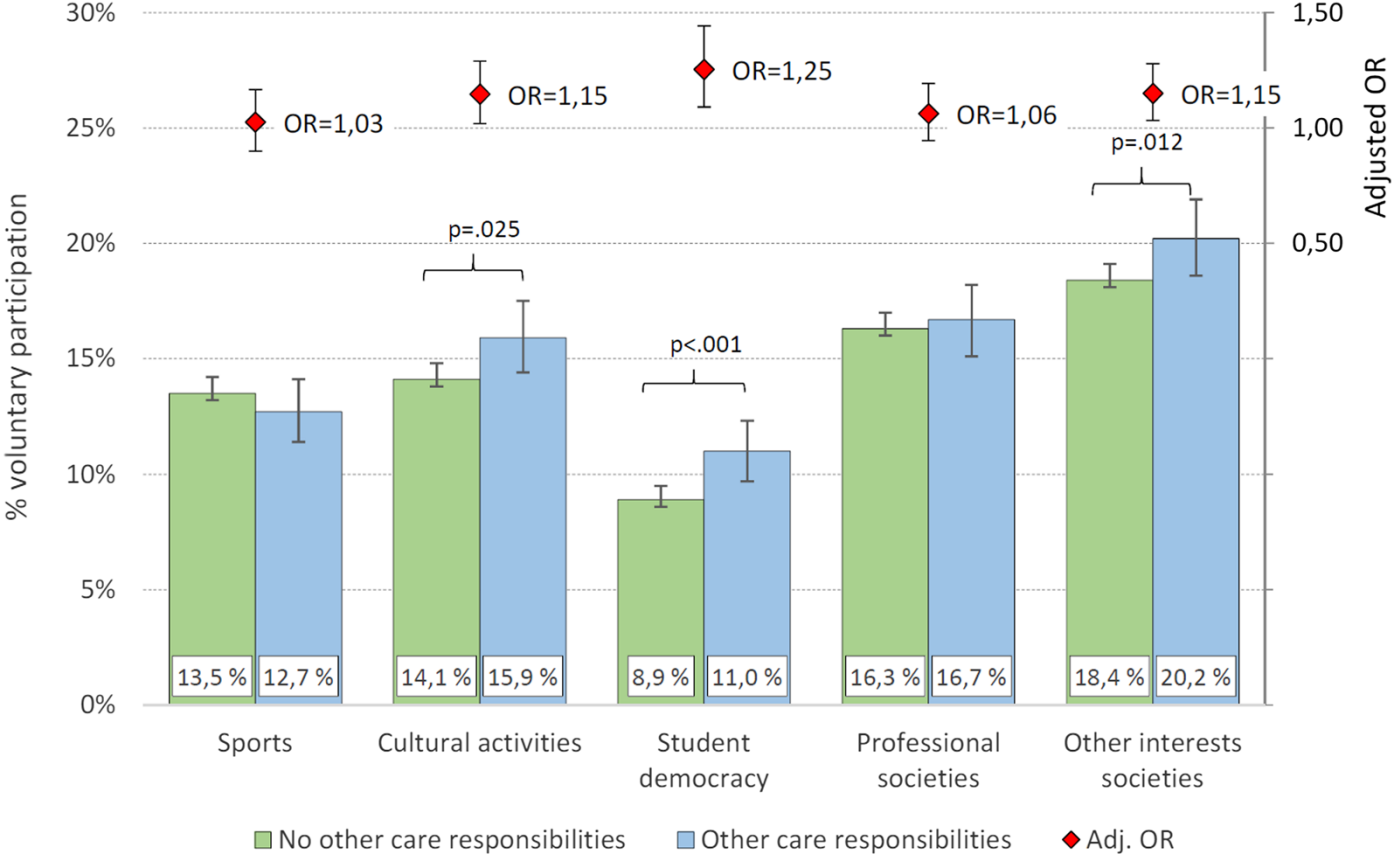
Participation in leisure activities in students with and without care responsibilities. Note. Error bars represent 95% confidence intervals

Providing informal care to others due to physical or mental illnesses, substance abuse, or disabilities was significantly associated with being less physically active. While 20.7% of student who did *not* have care responsibilities fulfilled the recommended criteria of 150 min of MVPA per week, the corresponding proportion was 17% among YACs (*p* < 0.001). A different pattern emerged for participation in organized volunteer student activities. As displayed in Fig. 1, there were no significant differences between students with and without care responsibilities regarding participation in sport activities. Students with care responsibilities were in fact more likely to be involved in both cultural activities (adj. OR = 1.15, 95% CI: 1.02–1.29), student democracy (adj. OR = 1.25, 95% CI 1.09–1.44), as well as other interests societies (adj. OR = 1.15, 95% CI 1.03–1.28), compared to students without care responsibilities (see Fig. 1 for details).

### Feelings of loneliness and number of close friends

**Fig. 2:**
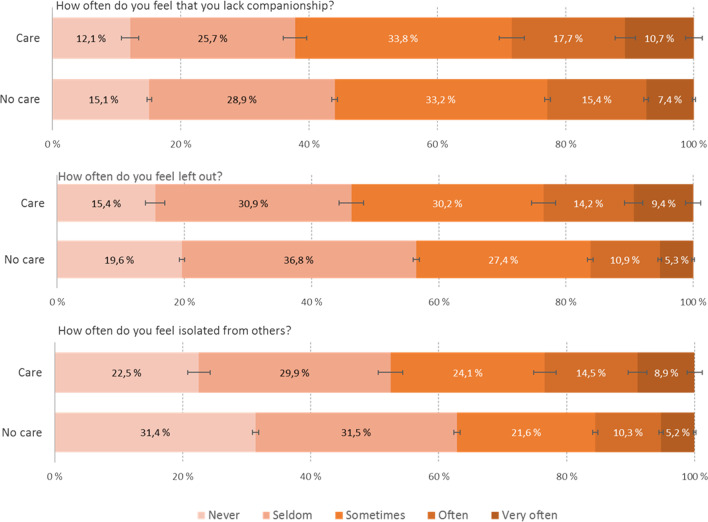
Response pattern of loneliness items among students with and without other care responsibilities. Note. Error bars represent 95% confidence intervals

Table 3 and Fig. 2 show that YACs to a larger extent report feeling of loneliness compared to their peers without care responsibilities. This pattern was evident across all three T-ILS items, with YACs more often reporting that they lacked companionship, felt left out, and felt isolated from others. While 5.3% of the control group reported “often” or “very often” on all these three items, the corresponding proportion in the YAC group was 8.0% (*p* < 0.001). There was also a statistically significant group difference in number of close friends, with YACs reporting having slightly fewer close friends than their peers (3.5 vs 3.6, *p* < 0.0019; see Table 3 and Fig. 2 for details).

### Hours of care during weekdays

**Fig. 3:**
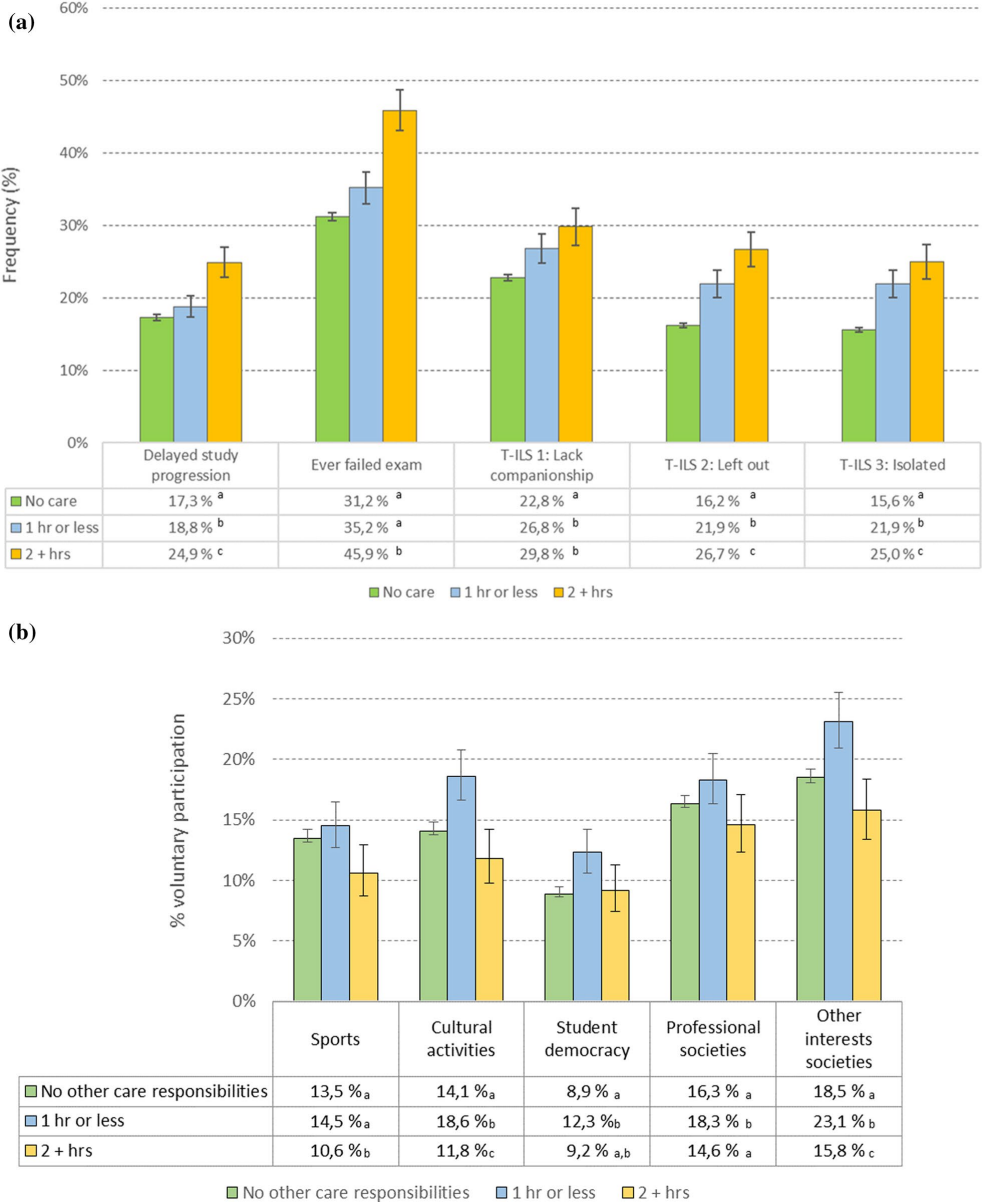
**a** Study progress and loneliness by hours of care responsibilities. Note. Error bars represent 95% confidence intervals. Values in the same column not sharing the same subscript (a,b,c) are significantly different at *p* < ,05 in the two-sided test of equality for column proportions. **b** Participation in organized volunteer student activities by hours of care responsibilities. Note. Error bars represent 95% confidence intervals. Values in the same column not sharing the same subscript (a,b,c) are significantly different at *p* < .05 in the two-sided test of equality for column proportions

We found delayed study progress to be significantly associated with the amount of care provided on weekdays in a dose–response manner. While 17.3% of students with no care responsibility reported delayed study progress, the corresponding proportion was 18.8% and 24.9% for those reporting ≤ 1 h and ≥ 2 h or more of daily caring (see Fig. 3a for details). Ever having failed exams was also dependent on hours of caring, with students spending ≥ 2 h being more likely to have failed exam(s) (45.9%) compared to students with no care (31.2%) and YACs with ≤ 1 h of care (35.2%). No difference in failed exams was found between students without care responsibility and YACs caring ≤ 1 h per day. A similar pattern was found for hours of studying per week, with no differences between students without care responsibility and YACs ≤ 1 h of caring per day (mean difference: − 0.53, [95% Cl − 1.39 to 0.32], *p* = 0.221), whereas students with ≥ 2 h of caring reported less hours of studying, both compared to those without care responsibility (mean difference: − 1.09, [95% Cl − 2.15 to − 0.03], *p* = . 045) and those with ≤ 1 h of caring per day (mean difference: − 1.62, [95% Cl − 2.96 to -0.28], *p* = . 018).

As displayed in Fig. 3a, a dose–response pattern was found between hours of caring and feelings of loneliness. YACs with ≤ 1 h of caring per day were more likely to feel left out and isolated compared to non-YACs, whereas YACs with ≥ 2 h of daily caring reported more loneliness than YACs with ≤ 1 h of daily care (all *p’*s < 0.05).

Hours of daily caring was also associated with leisure activities (Fig. 3b). Increased involvement in  recreational activities was the case for YACs reporting ≤ 1 h of daily caring, whereas YACs spending ≥ 2 h daily on caring were less involved in sports, cultural activities, and other interest societies compared both to non-YACs and YACs with ≤ 1 h of caring (*p* < 0.05). Also, YACs with ≥ 2 h were less involved in professional societies compared to those spending ≤ 1 h providing care per day.

## Discussion

The present study contributes to the limited research evidence on young adult carers (YACs) aged 18 to 25 years. A national population study of young adults in higher education (*N* = 40,203, 18–25 years) was examined, comparing students with and without informal care responsibility due to physical or mental illness, substance abuse, or disabilities in family members, relatives, or others. YACs reported poorer study progress compared to students without care responsibilities. Regarding recreational activities, caring responsibility differed depending on number of hours of caring on weekdays. Whereas YACs who provided 1 h or less of daily care were more likely to participate in voluntary student activities, those with 2 h or more of daily caring reported less involvement in such activities. YACs also reported less physical exercise, somewhat lower number of close friends, and more loneliness compared to students without care responsibilities. A dose response pattern was found, with number of hours providing care being associated with poorer study progress and more loneliness.

### Study progress

Findings of YACs having more delayed study progress (i.e., number of failed exams and changes in study program) correspond to negative educational consequences of caring (e.g., high degree of lateness and absence from school) reported in previous studies on YACs [6, 11, 35]. In line with Becker and Sempik [11], we found a dose–response pattern between caring and education, with greater caring responsibilities associated with more negative consequences. However, based on our findings we cannot account for the mechanisms in which informal caring influence education. Hours spent on studies per week was no different between non-carers and carers reporting 1 h or less of care per day on weekdays. This indicates that YACs with less care responsibility prioritized studies as much as their non-caring counterparts measured by hours spend studying. However, delayed study progression and failed exams among YACs with 2 h or more of caring per day may indicate conflicting demands between studies and caring. Thus, combination of lack of time and energy and the need to balance between care responsibilities and educational priorities are possible explanations for the association between caring responsibilities and poorer study progress, particularly for YACs who provide caring for several hours per day.

Poorer study progress among YACs with extensive care responsibility may also be the result of emotional challenges related to caring responsibility. Successful study progress necessitates continued focus and dedicated attention. Some YACs may be less concentrated on their studies, an assumption supported by a study by Blake-Holmes [35] who interviewed 20 carers (19–54 years) and found that their ability to engage with education was affected by tiredness, distraction, and worry about what was happening with the care-recipient at home. Similar findings have been reported by Stamatopoulos [10], describing sleep deprivation and worrying for the care-receiver as reasons for missing tests and assignment deadlines, as well as absenteeism and difficulties staying focused in class among young carers in secondary school. Hamilton and Adamson [18] found that caring responsibilities did not affect ambitions among YACs to go to university. Nevertheless, YACs choose courses and institutions based on what was possible or practical to combine within their caring responsibilities. Thus, caring responsibilities seemed to have priority, and perhaps also contribute to poorer study progress.

### Recreational activities

Compared to students without care responsibilities, YACs in the present study were more likely to participate in organized volunteer student activities (e.g., cultural activities, student democracy, and other interests societies). This may be explained by a previous study describing YACs to be involved in recreational activities if these activities were easily available on campus [6]. In our study increased recreational activities were reported only by YACs with 1 h or less of daily care responsibility, whereas YACs with more extensive care responsibility reported lesser involvement in leisure activities. This is in line with findings from a previous study where 66% of young carers (12–21 years) reported being unable to participate in activities outside of school hours because they were needed at home.

YACs reported being less physically active than students without care responsibilities. According to a systematic review on physical activity in informal carers, across age groups, informal carers place their own health needs as secondary to the health of the care-recipient [36]. Even though the number of studies examining physical activities among informal carers is very scarce, it  was concluded that the level of physical activity among informal carers is below what they themselves desire, below governmental guidelines and below what is commonly found in the general population. Furthermore, health issues often found among informal carers, such as bodily pain, anxiety and insomnia [15], are suggested as possible barriers towards physical activities in informal carers. Physical activity has been examined as a potential protective strategy against emotional exhaustion and burnout in carers, with results indicating that activities such as walking, meditation, climbing and yoga etc. may decrease distress, improve self-efficacy and sleep quality, and increase well-being within a wide age-range of informal caregivers [37]. Further studies are warranted examining how and to what degree interventions involving physical activity may affect the psychosocial and physical well-being of YACs.

### Loneliness and number of close friends

YACs in the current study reported more loneliness compared to their peers. Also, they reported having slightly fewer friends with whom they felt close to or could talk to. Feelings of being left out and isolated were more pronounced among YACs with more extensive care responsibility (2 h or more per day). According to Vasileiou, et al. [20] loneliness and social isolation is common among informal carers, probably due to restrictions in the caregiver situation, with carers having limited time and opportunity to build and maintain social relationships. However, loneliness may also be a result of powerlessness, helplessness, and feelings of overwhelming responsibility among carers [20]. Young carers report that they feel different from others and that others are not fully able to understand their situation or the challenges they are confronting [24]. Feelings of being more mature and different from their peers may limit YACs’ in establishing close friendships. Furthermore, fear of stigmatization and experiences of being bullied because of an ill or disabled family member, relative or friend have also been suggested to contribute to loneliness in young carers [10, 11]. As higher levels of social support has been related to positive outcomes among carers between 10 to 25 years [21], improving social support for YACs would probably reduce feelings of loneliness and increase positive health outcomes.

### Strengths and limitations

The present study comprise a large sample of YACs recruited from a national population study on students in higher education. The sample allows us to compare outcomes between young adults with and without care responsibilities. A strength of the present study is the use of psychometrically sound outcome measures. Furthermore, the study focuses on a group of young adults where little previous research has been published [1, 4, 5].

The YACs in the present study were identified by self-report. Because young carers are considered to be “a hidden group”, often not identified by professionals in health care, education, or social services [38], self-report measures are considered the best available strategy to identify this group of cares. Furthermore, the survey questions applied to identify YACs is considered to be reliable and has previously been evaluated as suitable to identify young carers [8].

However, limitations of the study include definition of YACs not provided for responders of the survey. This may have resulted in some YACs not being identified. On the other hand, as the concept of young carers or young adult carers are not commonly used in Norway, a definition could have resulted in responders with care responsibility not identifying themselves with this label. Furthermore, despite the large sample, the results should be interpreted in accordance with the relatively modest response rate for the survey (31%). Also, worth noticing is the fact that we have limited information about the students who did not participate in the survey.

YACs may experience barrier against entering higher education [6, 39]. Thus, an important selection bias is the exclusion of young adults who are not in higher education, but rather in training, employment, or receiving welfare benefits. Thus, the present findings are limited to YACs in higher education. As females constitute about 70% of the student population in Norwegian colleges/universities, the sex difference in our sample should not represent a substantial bias.

Study progress is probably related to cognitive abilities, and this could be considered a relevant confounder. However, most students have proved their academic skills to get into university. Furthermore, as reliable measures on IQ require resources that are hard to include in a large population study like the present, cognitive abilities were not controlled for in the analyses. Also, we find it unlikely that cognitive abilities or IQ are associated with young adults providing informal care, making it less relevant to include IQ as a confounding variable.

The present study did not test potential relationships across study progress, recreational activities, and loneliness, and possible pathways between the caring situation and functional impact. Hopefully future studies will include longitudinal designs where more complex models for prediction of outcomes in YACs may be evaluated.

Limitations regarding the measures used in this study needs to be commented. Ideally all three outcomes (study progress, recreational activities, and loneliness) should have been assessed more extensively, including a broader specter of variables within each domain. For example, whereas social relations were assessed by self-reported loneliness and number of close friends, well-known scales measuring social support and/or social network could have been applied. As large surveys like the present have limited space for extensive measures, we anticipate future studies that may replicate, dispute, or nuance the present findings.

As this was a cross-sectional study, we cannot determine the temporal order and causality between caring responsibilities and outcomes. However, caregiving most likely affects study progress, recreational activities, and social relations, rather than the other way around. Other limitations in the study include the lack of information about aspects of the caregiving, such as the type of care provided (e.g., emotional care, personal care), type of illness/disability in the care-recipient (e.g., mental health problems), the relationship to the care recipient (e.g., parent, sibling, friend), duration of the illness/disability, or duration of the caregiving. This is information needed to be included in future studies to further nuance our understanding of the consequences of informal caregiving for young adults.

### Implications

Young adulthood is a period characterized by increased independence, development of voluntary relationships, and decisions regarding education and career. Disruptions of these tasks can have long-term adverse effects. In some countries (e.g., UK, Australia) interest organizations for young carers below 18 years have been established, providing information, advice, support, social relations, practical skills, and referrals to specialist organizations, whereas other countries (e.g., Norway) have limited attention towards the needs of young carers, either above or below 18 years. Thus, increased attention and support for young people with care responsibilities is warranted within the health and educational systems. As the need of YACs to prioritize multiple demands and the lack of time to pursuit own interests may also be a barrier against receiving help and support, web-based interventions (e.g., for psychoeducation, social networking) have been suggested to make support more available [10]. Furthermore, as activities available on campus may be easier to attend for some YACs, psychosocial interventions, and/or physical activity directed at YACs could be offered as student activities. Counsellors and employees within the higher education system should be sensitized towards the needs of YACs. This could make it easier to recognize YACs and to help them balancing their caring responsibility and education (e.g., offer flexibility regarding exams, attendance, and study progress). When necessary, referral should be made to specialized services for the young carer him/herself and for the care-receiver (e.g., home-based care services, psychotherapy, physiotherapy). Finally, heightened public awareness and social appreciation of the contributions made by young adult caregivers may diminishing their sense of loneliness and isolation.

## Conclusion

The present study confirms that care responsibilities negatively affect important areas in the lives of young adult carers (i.e., study progress, physical activity, and feelings of loneliness). Recreational life (i.e., organized volunteer student activities) were negatively affected only for YACs with more extensive care responsibility. To limit the negative effects on the young adult carers, support attending to their needs must be established. Support should be made easily accessible and be a concern for both health- and educational organizations. The paucity of studies and the increasing need for informal caregivers suggest that further research is warranted to expand our knowledge on how to prevent negative outcomes for young adult careers. Hopefully future research will include longitudinal studies, allowing for analyses of mediating variables and thereby further increase our understanding of the associations between care responsibility and outcomes in YACs.

## Data Availability

The datasets for this article are not publicly available due to privacy regulations from the Norwegian Regional Committees for Medical and Health Research Ethics (REC). Requests to access the datasets should be directed to BS (borge.sivertsen@fhi.no). Guidelines for access to SHoT data are found at https: //www.fhi.no/en/more/access-to-data. Approval from the Regional Committee for Medical and Health Research Ethics (https://helseforskning.etikkom.no) is a pre-requirement.

## Abbreviations

YACs: Young adult carers
SHoT: Students’ Health and Wellbeing Study
